# The Tumor Microenvironment and Immune Response in Breast Cancer

**DOI:** 10.3390/ijms25020914

**Published:** 2024-01-11

**Authors:** Behjatolah Monzavi-Karbassi, Thomas Kelly, Steven R. Post

**Affiliations:** Department of Pathology, University of Arkansas for Medical Sciences, Little Rock, AR 72205, USA; karbassi@uams.edu (B.M.-K.); kellythomasj@uams.edu (T.K.)

## 1. Introduction

The complex interactions between cancer cells and their surrounding microenvironment are fundamental in determining tumor progression, response to therapy, and, ultimately, patient prognosis. Interactions within the tumor microenvironment (TME) can enhance or inhibit the efficacy of various treatments, including immunotherapies such as checkpoint inhibitors (CPIs) and CAR-T cell approaches [1,2,3,4,5]. The success of immunotherapy depends on the dynamic interactions within the TME, encompassing aspects of immune cells such as their spatial distribution, phenotypic composition, and functional state. Recently, there have been significant advances in characterizing the TME; however, an understanding of how interactions in the TME contribute to an effective antitumor immune response is incomplete.

The articles in this Special Issue address recent advances in in targeting the interactions between cancer cells and their microenvironment to formulate innovative therapeutic strategies. Mainstream and emerging research concepts concerning the TME are highlighted, emphasizing immune-targeted therapeutic strategies for treating breast cancer patients to encourage innovative inquiries and broaden the conversation in this area.

## 2. Immune Targeting of Tumor Cells

A fundamental issue in treating breast cancer patients relates to the availability of or, more precisely, the lack of therapeutics that specifically target cancer cells. This lack of cancer specificity is generally associated with a low therapeutic index, resulting in diminished treatment efficacy and a high incidence of undesirable side effects resulting from off-target toxicity. Thus, identifying cancer-specific drug targets is an area of intense research interest. The papers presented in this Special Issue illustrate the therapeutic potential of directly targeting tumor-specific antigens.

It has long been recognized that the malignant transformation of cancer cells results in the expression of molecules that are generally absent or present in low abundance in non-malignant cells. These molecules can be antigenic and elicit a cancer-specific immune response. Because of their cancer specificity, there continues to be significant interest in innovative strategies that target these molecules [6,7,8,9]. Active and passive immunization with targeted cancer vaccines and monoclonal antibodies are exciting approaches. The potency of these approaches is intrinsically tied to the tumor specificity and functional relevance of the targeted antigen. For example, the report by Loukinov et al. [10] describes a cancer vaccine developed against the Brother of the Regulator of Imprinted Sites (BORIS). As noted by the authors, BORIS is aberrantly and selectively expressed in various tumor types and, except for the testis, not in normal tissues, making it an excellent target for cancer vaccine development [11,12]. The results of their study using a rat model of breast cancer demonstrate that immunizing with a vaccine to BORIS suppresses the growth of breast tumors, with the cancer disappearing in a large portion of the treated animals. Tumor growth inhibition was associated with a BORIS-specific immune response and protection against rechallenge with tumor cells.

Chondroitin sulfate proteoglycan 4 (CSPG4) is a tumor-associated antigen aberrantly expressed by cancer cells [13]. Previous studies demonstrated the specificity of CSPG4 expression in triple-negative breast cancer and the role of sulfated chondroitin in the aggressive behavior of breast cancer cells in vitro [14,15]. Rather than using a vaccination approach, the study by Nounamo et al. [16] describes the therapeutic potential of an anti-CSPG4 monoclonal antibody (Mab VT68.2). Their study demonstrates that tumor-associated glycans mediate the binding of Mab VT68.2 to breast cancer cells and that this binding reduces both the viability and metastatic potential of these cells in vitro. These results highlight the therapeutic potential of mAbs directed at tumor-specific antigens.

Although primarily focused on diagnostics, the strategy proposed by Ferdinandov et al. [17] paves the way for reverse engineering approaches to create potent antitumor immunogens. This proof-of-principle study used a graph representation approach to characterize the reactivities of a repertoire of immunoglobulin M (IgM) in sera of cancer patients. Their results indicate that the peptide reactivity profiles of IgM can be used to distinguish between patients in different diagnostic groups. This knowledge could be leveraged to identify patients and develop therapies that effectively modulate the immune response to target tumor cells.

## 3. Deciphering Immune Regulatory Mechanisms

Identifying and understanding the many factors contributing to the immune response in breast cancer is key to developing novel immune-targeted therapeutics. These factors include cancer-cell-specific immune targets, such as the antigens described above, and factors in the TME, such as cytokines, that act on immune cells.

The pivotal role of the IL-33/ST2 axis in the TME is underscored in the article by Stojanovic et al. [18]. This review describes the multifaceted roles of IL-33/ST2 signaling in modulating inflammatory responses and its implications in breast cancer progression. IL-33 binds to the ST2L receptor to activate molecular events and signaling pathways that ultimately drive protumor activities, including modulating cancer cell proliferation, developing an antitumor immune response, and therapeutic resistance. Thus, there is considerable interest in targeting the IL-33/ST2 axis in treating breast cancer. The potential benefits and limitations of targeting this axis are discussed.

The review by Kumari et al. [19] provides a comprehensive overview of the butyrophilin and butyrophilin-like (BTN/BTNL) molecules and their potential as novel therapeutic targets to stimulate specific T-cell populations in the TME. Understanding the specific roles and mechanisms of BTN/BTNL molecules in this context could identify novel targets for therapeutic intervention, potentially enhancing the efficacy of existing immunotherapies or paving the way for the development of new therapeutic strategies.

## 4. Utilizing Systems Immunology

Systems biology approaches focusing on the interactions between cancer and immune cells offer a comprehensive perspective on the tumor microenvironment [20,21]. Techniques like single-cell sequencing have revealed the diversity of cell types and their functional state in the TME, thus providing novel insights into tumor immunology. Computational models are being developed to simulate these interactions and predict responses to therapeutic approaches such as immunotherapy. This integrated approach aids in understanding tumor immunology, identifying potential targets for therapy, and designing more effective cancer treatments.

Koelsh and Manjili [22] describe a systems immunology approach to unravel the intricacy of the TME. They focus on the elaborate network through which immune cells orchestrate their interactions and responses against cancer. They reason that a reductionist perspective, while valuable, may only partially encapsulate the complicated and dynamic nature of the TME. Instead, they advocate for a more integrative approach to understanding the complexities of the TME. These systems biology approaches integrate advanced multiomics technologies, such as single-cell RNA sequencing (scRNA-seq), to reveal networks and consequences of cellular interactions. This could identify immunologic patterns in the TME and accelerate the discovery of novel therapeutic targets and strategies that could pivotally influence breast cancer treatment.

## 5. Conclusions

This Special Issue highlights some of the expanding progress in understanding the role of the TME in breast cancer and the importance of combining cutting-edge molecular screening approaches and analytical methods to delineate and understand the complex interactions of the TME. Hopefully, the information presented will catalyze continued collaborative efforts and pioneering research that pave the way for breakthroughs in cancer therapy.

## References

[1] Kotsifaki A., Alevizopoulos N., Dimopoulou V., Armakolas A. (2023). Unveiling the Immune Microenvironment’s Role in Breast Cancer: A Glimpse into Promising Frontiers. Int. J. Mol. Sci..

[2] Zhang J., Huang D., Saw P.E., Song E. (2022). Turning cold tumors hot: From molecular mechanisms to clinical applications. Trends Immunol..

[3] Iglesias-Escudero M., Arias-Gonzalez N., Martinez-Caceres E. (2023). Regulatory cells and the effect of cancer immunotherapy. Mol. Cancer.

[4] Yi M., Li T., Niu M., Mei Q., Zhao B., Chu Q., Dai Z., Wu K. (2023). Exploiting innate immunity for cancer immunotherapy. Mol. Cancer.

[5] Liu Y., Hu Y., Xue J., Li J., Yi J., Bu J., Zhang Z., Qiu P., Gu X. (2023). Advances in immunotherapy for triple-negative breast cancer. Mol. Cancer.

[6] Tan Y., Chen H., Gou X., Fan Q., Chen J. (2023). Tumor vaccines: Toward multidimensional anti-tumor therapies. Hum. Vaccines Immunother..

[7] Zefferino R., Conese M. (2023). A Vaccine against Cancer: Can There Be a Possible Strategy to Face the Challenge? Possible Targets and Paradoxical Effects. Vaccines.

[8] Vajari M.K., Sanaei M.J., Salari S., Rezvani A., Ravari M.S., Bashash D. (2023). Breast cancer vaccination: Latest advances with an analytical focus on clinical trials. Int. Immunopharmacol..

[9] Schumacher T.N., Schreiber R.D. (2015). Neoantigens in cancer immunotherapy. Science.

[10] Loukinov D., Anderson A.L., Mkrtichyan M., Ghochikyan A., Rivero-Hinojosa S., Tucker J., Lobanenkov V., Agadjanyan M.G., Nelson E.L. (2023). A Therapeutic Vaccine Targeting Rat BORIS (CTCFL) for the Treatment of Rat Breast Cancer Tumors. Int. J. Mol. Sci..

[11] Loukinov D. (2018). Targeting CTCFL/BORIS for the immunotherapy of cancer. Cancer Immunol. Immunother. CII.

[12] Mkrtichyan M., Ghochikyan A., Davtyan H., Movsesyan N., Loukinov D., Lobanenkov V., Cribbs D.H., Laust A.K., Nelson E.L., Agadjanyan M.G. (2011). Cancer-testis antigen, BORIS based vaccine delivered by dendritic cells is extremely effective against a very aggressive and highly metastatic mouse mammary carcinoma. Cell Immunol..

[13] Ilieva K.M., Cheung A., Mele S., Chiaruttini G., Crescioli S., Griffin M., Nakamura M., Spicer J.F., Tsoka S., Lacy K.E. (2017). Chondroitin Sulfate Proteoglycan 4 and Its Potential As an Antibody Immunotherapy Target across Different Tumor Types. Front. Immunol..

[14] Cooney C.A., Jousheghany F., Yao-Borengasser A., Phanavanh B., Gomes T., Kieber-Emmons A.M., Siegel E.R., Suva L.J., Ferrone S., Kieber-Emmons T. (2011). Chondroitin sulfates play a major role in breast cancer metastasis: A role for CSPG4 and CHST11 gene expression in forming surface P-selectin ligands in aggressive breast cancer cells. Breast Cancer Res..

[15] Behrens A., Jousheghany F., Yao-Borengasser A., Siegel E.R., Kieber-Emmons T., Monzavi-Karbassi B. (2020). Carbohydrate (Chondroitin 4) Sulfotransferase-11-Mediated Induction of Epithelial-Mesenchymal Transition and Generation of Cancer Stem Cells. Pharmacology.

[16] Nounamo B., Jousheghany F., Siegel E.R., Post S.R., Kelly T., Ferrone S., Kieber-Emmons T., Monzavi-Karbassi B. (2023). VT68.2: An Antibody to Chondroitin Sulfate Proteoglycan 4 (CSPG4) Displays Reactivity against a Tumor-Associated Carbohydrate Antigen. Int. J. Mol. Sci..

[17] Ferdinandov D., Kostov V., Hadzhieva M., Shivarov V., Petrov P., Bussarsky A., Pashov A.D. (2023). Reactivity Graph Yields Interpretable IgM Repertoire Signatures as Potential Tumor Biomarkers. Int. J. Mol. Sci..

[18] Stojanovic B., Gajovic N., Jurisevic M., Stojanovic M.D., Jovanovic M., Jovanovic I., Stojanovic B.S., Milosevic B. (2023). Decoding the IL-33/ST2 Axis: Its Impact on the Immune Landscape of Breast Cancer. Int. J. Mol. Sci..

[19] Kumari R., Hosseini E.S., Warrington K.E., Milonas T., Payne K.K. (2023). Butyrophilins: Dynamic Regulators of Protective T Cell Immunity in Cancer. Int. J. Mol. Sci..

[20] Akhoundova D., Rubin M.A. (2022). Clinical application of advanced multi-omics tumor profiling: Shaping precision oncology of the future. Cancer Cell.

[21] Baysoy A., Bai Z., Satija R., Fan R. (2023). The technological landscape and applications of single-cell multi-omics. Nat. Rev. Mol. Cell Biol..

[22] Koelsch N., Manjili M.H. (2023). From Reductionistic Approach to Systems Immunology Approach for the Understanding of Tumor Microenvironment. Int. J. Mol. Sci..

